# Exploring shared molecular signatures and regulatory mechanisms in nonalcoholic steatohepatitis and inflammatory bowel disease using integrative bioinformatics analysis

**DOI:** 10.1038/s41598-024-62310-w

**Published:** 2024-05-27

**Authors:** Zixuan Zhong, Minxuan Xu, Chenxu Ge, Jun Tan

**Affiliations:** 1 Chongqing Key Laboratory of Medicinal Resources in the Three Gorges Reservoir Region, School of Biological and Chemical Engineering, Chongqing University of Education, Chongqing, 400067 People’s Republic of China; 2 Research Center of Brain Intellectual Promotion and Development for Children Aged 0-6 Years, Chongqing University of Education, Chongqing, 400067 People’s Republic of China; 3 Key Laboratory of Biorheological Science and Technology (Chongqing University), Ministry of Education, College of Bioengineering, Chongqing University, Chongqing, 400030 People’s Republic of China

**Keywords:** Non-alcoholic steatohepatitis (NASH), Inflammatory bowel disease (IBD), WGCNA, Shared signature genes, Diagnostic validity, Single-cell analysis, Genetic markers, Mechanisms of disease

## Abstract

The co-existence of inflammatory bowel disease (IBD) and non-alcoholic steatohepatitis (NASH) has raised interest in identifying shared molecular mechanisms and potential therapeutic targets. However, the relationship between these two diseases remains unclear and effective medical treatments are still lacking. Through the bioinformatics analysis in this study, 116 shared differentially expressed genes (SDEGs) were identified between IBD and NASH datasets. GO and KEGG pathway analyses revealed significant involvement of SDEGs in apoptotic processes, cell death, defense response, cytokine and chemokine activity, and signaling pathways. Furthermore, weighted gene co-expression network analysis (WGCNA) identified five shared signature genes associated specifically with IBD and NASH, they were CXCL9, GIMAP2, ADAMTS5, GRAP, and PRF1. These five genes represented potential diagnostic biomarkers for distinguishing patients with diseases from healthy individuals by using two classifier algorithms and were positively related to autophagy, ferroptosis, angiogenesis, and immune checkpoint factors in the two diseases. Additionally, single-cell analysis of IBD and NASH samples highlighted the expression of regulatory genes in various immune cell subtypes, emphasizing their significance in disease pathogenesis. Our work elucidated the shared signature genes and regulatory mechanisms of IBD and NASH, which could provide new potential therapies for patients with IBD and NASH.

## Introduction

Non-alcoholic fatty liver disease (NAFLD) is a prevalent liver disorder closely related to insulin resistance, obesity, and metabolic syndrome^1,2^. It encompasses a spectrum of conditions ranging from simple steatosis to non-alcoholic steatohepatitis (NASH). NASH is characterized by fat accumulation, inflammation, and fibrosis, which can progress to cirrhosis, hepatic failure, and hepatocellular carcinoma (HCC)^3–5^. The activation of innate immunity in the liver is a pivotal factor in triggering and promoting the inflammatory response, driving the progression of NAFLD to NASH and probably contributing to liver fibrosis in NASH^6^.

Inflammatory Bowel Disease (IBD), including ulcerative colitis (UC) and Crohn’s disease (CD), is a chronic inflammatory condition primarily targeting the gastrointestinal tract. The intricate interplay involving the genetic factors, immune system dysregulation, environmental influences, and microbial components results in persistent intestinal inflammation of IBD^7,8^. IBD may serve as a precursor to various complex ailments, including hepatic steatosis, hepatic amyloidosis, sclerosing cholangitis, autoimmune hepatitis, and liver abscess^9–11^. Additionally, the presence of obesity and overweight conditions, observed in NAFLD and NASH, is also prevalent among individuals diagnosed with IBD^12–14^.

Recent studies have demonstrated an elevated prevalence of NAFLD and NASH within the IBD patients cohort, with reported incidences ranging from 8 to 59%^15–22^. Despite the lack of complete understanding of this association, a recent retrospective study conducted by Sourianarayanane et al*.* confirmed that approximately one-third of individuals diagnosed with IBD globally have developed NAFLD, with a two-fold increased risk of NAFLD occurrence in IBD patients compared to those without IBD^16^. Furthermore, Bessissow et al.^20^ found that the emergence of incident NAFLD is a common occurrence among IBD patients, and these individuals may subsequently progress to an advanced state of liver fibrosis characteristic of NASH. Magrì et al*.* suggested that metabolic syndrome serves as a specific risk factor for advanced liver fibrosis among IBD patients concurrently afflicted with NAFLD^22^. Besides the metabolic syndrome, IBD-specific risk factors encompass malnutrition, intestinal inflammation, steroid exposure, drug-induced hepatotoxicity, and gut dysbiosis, all potentially contribute to the pathogenesis of NASH^8,15,16^. However, further studies are required to clarify these risk factors. Although accumulating evidence suggests that the pathogenesis of both NASH and IBD involves intricate interplay among genetic predisposition, immune system dysfunction, environmental factors, and metabolic disorders^7,23–28^, the biomolecular mechanisms and crucial signaling molecules commonly shared by NASH and IBD remain unknown.

In this study, we aimed to elucidate the shared genetic markers and key molecular pathways that contribute to the progression of NASH and IBD by employing advanced computational methods and integrated bioinformatics analyses. Our findings may uncover the potential connection and shared molecular signatures between NASH and IBD, offering insights into the pathophysiology of both diseases and potentially paving the way for novel therapeutic strategies benefiting patients with overlapping conditions of NASH and IBD.

## Materials and methods

### Data collection

We searched the GEO database (https://www.ncbi.nlm.nih.gov/geo/, Access Date: 2023.05) using the terms “Inflammatory Bowel Disease” and “Nonalcoholic Steatohepatitis” to identify gene expression profiles associated with NASH. The following criteria were applied as filters to ensure data quality and reliability: (1) Inclusion of array-based expression profiling or high-throughput mRNA sequencing. (2) Inclusion of both case and control groups in all datasets, with each group containing no fewer than 6 samples. (3) The number of samples in each group was required to be a minimum of 10 for WGCNA analysis. Finally, the four GEO datasets GSE59071, GSE36807, GSE89632, and GSE164760 were selected.

GSE59071 and GSE36807 are IBD datasets, while GSE89632 and GSE164760 are NASH datasets. The GSE59071 dataset included 116 samples, of which 8 active CD samples, 74 active UC samples, and 11 healthy samples were used in this study. The GSE36807 datasets included 35 samples, of which 13 CD samples, 15 UC samples, and 7 healthy samples were used in this study. The GSE89632 dataset included 63 samples, of which 19 NASH samples and 24 healthy samples were used in this study. The GSE164760 dataset included 170 samples, of which 74 NASH samples and 6 healthy samples were used in this study. The GSE164985 and GSE190487 datasets were also downloaded from GEO database for single-cell analysis. The specific information of all datasets was detailed in Table 1.

**Table 1:** Detailed information of the six GEO datasets containing patients with NASH and IBD.

ID	GSE number	Samples	Organism	Disease	Group
1	GSE59071	82 patients and 11 controls	Homo sapiens	IBD	SWDEGs analysis
2	GSE89632	19 patients and 24 controls	Homo sapiens	NASH	SWDEGs analysis
3	GSE36807	28 patients and 7 controls	Homo sapiens	IBD	Validation
4	GSE164760	74 patients and 6 controls	Homo sapiens	NASH	Validation
5	GSE164985	–	Homo sapiens	IBD	Single-cell analysis
6	GSE190487	–	Homo sapiens	NAFLD/NASH	Single-cell analysis

### Identification of differentially expressed genes

To identify the common genetic effects of NASH and IBD, the ‘limma’ (version 3.44.3) R package with *P* value < 0.05 and |Fold change (FC)|> 1.5 were used to explore the differentially expressed genes (DEGs) in GSE59071 and GSE89632. Then, the DEGs of NASH and IBD were visualized using the ‘complexheatmap’ (version 3.1.2) and ‘ggplot2’ (version 3.3.3) R packages to generate the heat maps and volcano maps, respectively. The overlapping DEGs in NASH and IBD were calculated using R-language, and these shared differentially expressed genes (SDEGs) with consistent up-regulated or down-regulated trends were retained for subsequent analysis.

### Analysis of functional classification and pathway enrichment

To obtain the common biological functions and signaling pathways underpinning the initiation and progression of the two diseases, the SDEGs between the IBD dataset GSE59071 and NASH dataset GSE89632 were analyzed by Gene Ontology (GO) and Kyoto Encyclopedia of Genes and Genomes (KEGG)^12^ pathway enrichment using “ClusterProfiler” (version3.16.1), ‘org.Hs.eg.Db’(version 3.11.4), and ‘GOplot’ (version 1.0.2) R packages, *P* value < 0.05 was set for enrichment.

### Protein–protein interaction network analysis

The STRING database (http://string-db.org, Access Date: 2023.06) was employed to predict and delineate potential protein interactions, thereby facilitating the construction of a protein–protein interaction (PPI) network. Within this network, PPI pairs with a reliability score > 0.4 were considered to be statistically significant. Cytoscape (version 3.8.0) was used for visualizing molecular interaction networks. Subsequently, the identification of pivotal hub genes, characterized by top ten genes with highest connectivity within the PPI networks, was conducted employing the Maximal Clique Centrality (MCC) method through the cytoHubba plugin within the Cytoscape platform. Furthermore, to identify and delineate the most influential clusters within the PPI network, we applied the Molecular Complex Detection (MCODE) plugin available in Cytoscape software.

### WGCNA for co-expression network construction

To obtain WGCNA modules of NASH and IBD, the ‘WGCNA’ (version 1.70.3) R package^29^ was applied to the IBD dataset GSE59071 and NASH dataset GSE89632. First, the variance for each gene expression value was calculated and a filtering process was employed to eliminate genes exhibiting absolute deviations exceeding 25% in relation to the median value. Furthermore, potential outlier samples were addressed by employing the “hclust” function for hierarchical clustering analysis. Samples exhibiting outlier characteristics were excluded using the ‘goodSampleGenes’ function (Supplementary Fig. 1A, B). To construct scale-free networks, β value was evaluated using the “pickSoftThreshold” function and a soft threshold β of 14 was identified as suitable for both NASH and IBD datasets (Supplementary Fig. 2A, B). Subsequently, hierarchical clustering dendrograms were constructed and similar genes were grouped into distinct modules, with a minimum of 30 genes per module. Analogous modules were consolidated based on a Module Eigengenes Dissimilarity Threshold (MEDissThres) = 0.2. Finally, a Pearson correlation analysis was conducted to establish the association between modules and the specific disease phenotypes of interest. Our analysis focused on modules demonstrating remarkable correlations with the targeted phenotypic attributes. From these disease-related modules, genes were selected for subsequent analysis.

### Identification of SWDEGs in NASH and IBD

Firstly, the hub modules displaying strong association with NASH and IBD were screened out (absolute correlation coefficient $\ge \hspace{0.17em}$0.6 and *P* value < 0.001) based on the module trait correlation and the significance levels of eigengenes in relation to phenotypic traits within each module. Then, the shared genes in hub modules positively related to the two diseases and up-regulated SDEGs were identified using the Jvenn online tool (http://jvenn.toulouse.inra.fr/app/example.html, Access Date: 2023.06)^30^, and these genes were considered as shared gene signatures (SWDEGs). Finally, the expression levels of SWDEGs between patients with IBD and normal controls in validation datasets GSE36807, NASH and normal controls in validation datasets GSE164760 were confirmed and represented using box plots respectively.

### Validation of diagnostic capacity of SWDEGs for NASH and IBD

Two classification algorithms including support vector machine (SVM)^31^ and logistic regression (LR)^32^ were employed to assess the diagnostic validity of SWDEGs for NASH and IBD. Both algorithms are widely used methods for binary classification problems. The area under the ROC curve (AUC) was calculated based on SWDEGs expression in GSE59071, GSE89632, GSE36807, and GSE164760, respectively using ‘pROC’ (version 1.18.0), ‘e1071’ (version 1.7.9), and ‘rms’ (version 6.2.0) R packages. The training set obtained 70% of the total samples, and the test set obtained 30% of the total samples for two classifier algorithms.

### Transcription factors and miRNAs analysis

The SWDEGs in NASH and IBD have been considered in this analysis. The miRNAs-SWDEGs interaction networks and transcription factors (TFs)–SWDEGs interaction networks were identified using Network Analyst (https://www.networkanalyst.ca/, Access Date: 2023.07)^33^. TarBase v.8 database (https://dianalab.e-ce.uth.gr/html/diana/web/index.php?r=tarbasev8, Access Date: 2023.07) was used for discovering miRNAs-DEGs interaction networks^34^, and JASPAR 2022 (https://jaspar.genereg.net/, Access Date: 2023.07) was used for TFs–SWDEGs interaction network analysis^35^. Both TFs–SWDEGs and miRNAs–SWDEGs interaction networks were visualized in Cytoscape.

### Correlation analysis of SWDEGs with ferroptosis, autophagy, angiogenesis, and immune

A total of 304 ferroptosis-related genes (FRGs) were obtained from the FerrDb V2 database (http://www.zhounan.org/ferrdb/current/, Access Date: 2023.07), 222 autophagy-related genes (AURGs) were obtained from HADb (Human Autophagy Database, http://www.autophagy.lu/index.html, Access Date: 2023.07), 36 angiogenesis-related genes (ANRGs) were obtained from the MSigDB Team (Hallmark Gene set) and 79 immune checkpoint-related genes (ICRGs) according to a previous paper^36^. Pearson’s correlation analysis was used to reveal the relationships between SWDEGs and four gene sets (FRGs, AURGs, ANRGs, and ICRGs). Then, the top 10 genes of high correlation values with SWDEGs in each gene set were extracted and visualized them using ChiPlot (https://www.chiplot.online/, Access Date: 2023.07).

### Single‑cell sequencing analysis

The GSE164985 and GSE190487 datasets were first processed using the ‘Seurat’ (version 4.0.2) R package^16^, and then UMAP method analysis was employed to identify the spatial associations among the various clusters present within the datasets. 'SingleR' (version 1.2.4) and 'celldex' (version 1.11.1) R packages were utilized to provide coprehensive annotation^37^. In tandem, we performed a manual validation of cell type annotation utilizing CellMarker (http://xteam.xbio.top/CellMarker/, Access Date: 2023.07). The marker gene identification for each distinct cell subtype was achieved through the application of the FindAllMarkers function, employing a log-fold change (logfc) threshold of 0.25. Genes with *P* value < 0.05 were chosen as separate marker genes for each cell cluster (Supplementary Table S1).

### Collection of human clinical samples

Human liver tissue samples of NASH group (n = 5) and non-steatosis group (n = 5) used in the current study were collected from adult patients as our previously reported^38^. The liver samples with a NASH activity score (NAS) of 0 were classified as non-steatosis group, and samples with a NAS ≥ 5 or a NAS of 3–4 but showing fibrosis were included in the NASH group. All procedures associated with human subjects used in this study were based on the Declaration of Helsinki, and completely approved by the Academic Research Ethics Committee in Chongqing Key Laboratory of Medicinal Resources in the Three Gorges Reservoir Region and other participating units. According to the research approved by IRB/IEC, written informed consents were required and samples were then collected from the donors.

Human colon tissue samples from patients with CD or UC were obtained from the Department of Gastrointestinal Surgery and the Clinical Trial Research Center at Shandong Cancer Hospital and Institute. The diagnosis of UC or CD was confirmed through radiological, clinical, and endoscopic evaluations, and histological data analysis. The samples with and without UC or CD phenotype were categorized into the IBD group (n = 5) and non-IBD group (n = 5), respectively. All patients and donors provided informed consent for the use of their samples in this study, which was approved by the Institutional Research Ethics Committees of Shandong First Medical University & Shandong Academy of Medical Sciences and Chongqing University of Education (Ethics Approval Number: CQUE202014EA02).

### Immunofluorescence examination

Immunofluorescence analysis was carried out as described previously^38,39^. In brief, 5 µm thick frozen tissue sections were placed for 20 min at room temperature and then washed with PBS for three times. The tissue sections were blocked using a solution containing 10% goat serum (Beyotime) and 0.3% Triton X-100 (Beyotime) for 1 h at room temperature and then incubated with primary antibodies against CXCL9, GIMAP2, ADAMTS5, GRAP, PRF1 and F4-80 (Invitrogen) at 4 °C overnight in the indicated groups. After three times of PBS washes, the tissue slides were incubated with the corresponding goat anti-mouse or anti-rabbit IgG antibodies (Abcam) at room temperature in the dark for 1 h. Finally, the sections were stained with 2-(4-Amidinophenyl)-6-indole-carbamidine dihydrochloride (DAPI, Beyotime) solution for nuclei staining. All the histological procedure was performed in accordance with the standard procedures as indicated in reagent specifications. Images were visualized and captured under fluorescence microscopy (Olympus, Japan).

### Statistical analysis

Statistical analysis was performed using R programming language (version 4.0.5). The PPI network, miRNAs-SWDEGs and TFs-SWDEGs interaction networks were visualized in Cytoscape (version 3.8.0). A threshold of *P* value < 0.05 was considered to indicate statistical significance.

### Ethics statement

The studies involving human participants were approved by the Academic Research Ethics Committee in Chongqing Key Laboratory of Medicinal Resources in the Three Gorges Reservoir Region and Chongqing University of Education.

## Results

### Identification of common DEGs between NASH and IBD

**Figure 1:**
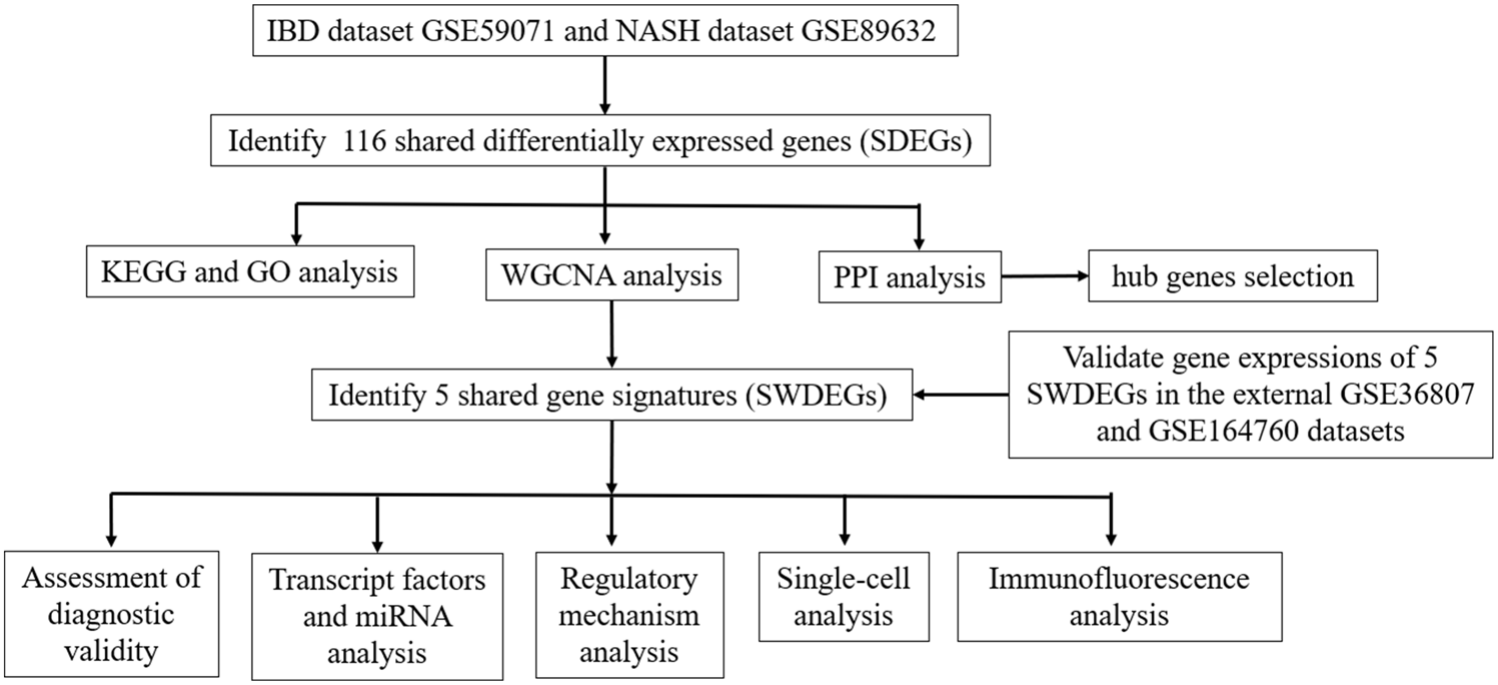
Flow chart of this study.

**Figure 2:**
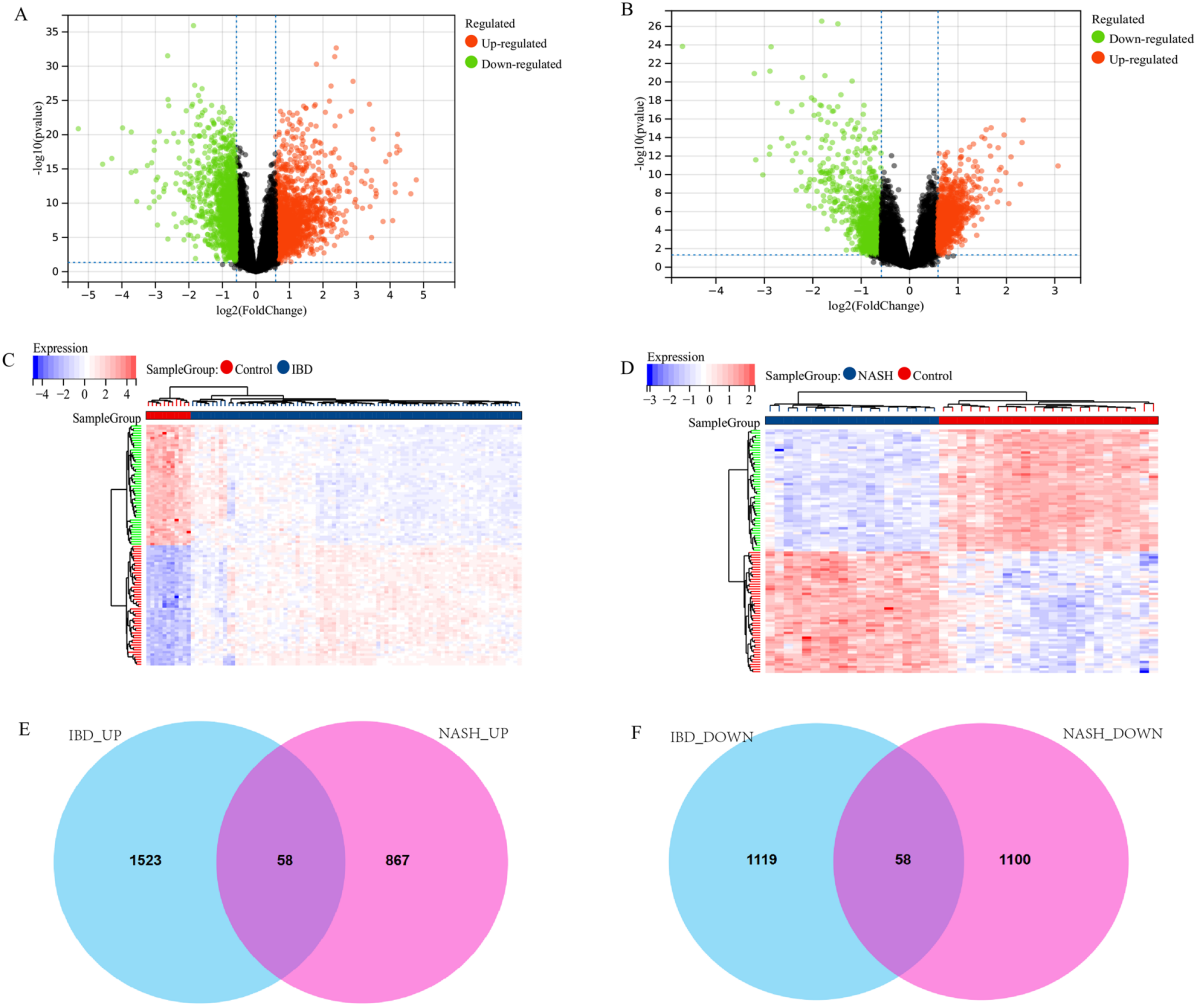
Identification and analysis of shared DEGs (SDEGs) in IBD dataset GSE59071 and NASH dataset GSE89632. (**A**) Volcano plot of the DEGs in IBD. (**B**) Volcano plot of the DEGs in NASH. (**C**) Heatmap of DEGs in IBD. (**D**) Heatmap of DEGs in NASH. (**E**) Venn diagram of up-regulated SDEGs in NASH and IBD. (**F**) Venn diagram of down-regulated SDEGs in NASH and IBD.

The workflow of the study was displayed in Fig. 1. The IBD dataset GSE59071 and NASH dataset GSE89632 were downloaded from the NCBI GEO database, and 1581 up-regulated and 1177 down-regulated DEGs were identified in the GSE59071 dataset, 925 up-regulated and 1158 down-regulated DEGs were identified in GSE89632 with *P* value < 0.05 and |Fold change|> 1.5. The visualization of DEGs within the two datasets was facilitated through the utilization of volcano plots and heatmap analyses (Fig. 2A–D). According to the result, 116 shared DEGs (SDEGs) between GSE59071 and GSE89632 were identified (Fig. 2E,F), in which 58 SDEGs were up-regulated and 58 SDEGs were down-regulated in NASH and IBD datasets.

### GO and KEGG pathway analysis of SDEGs

**Figure 3:**
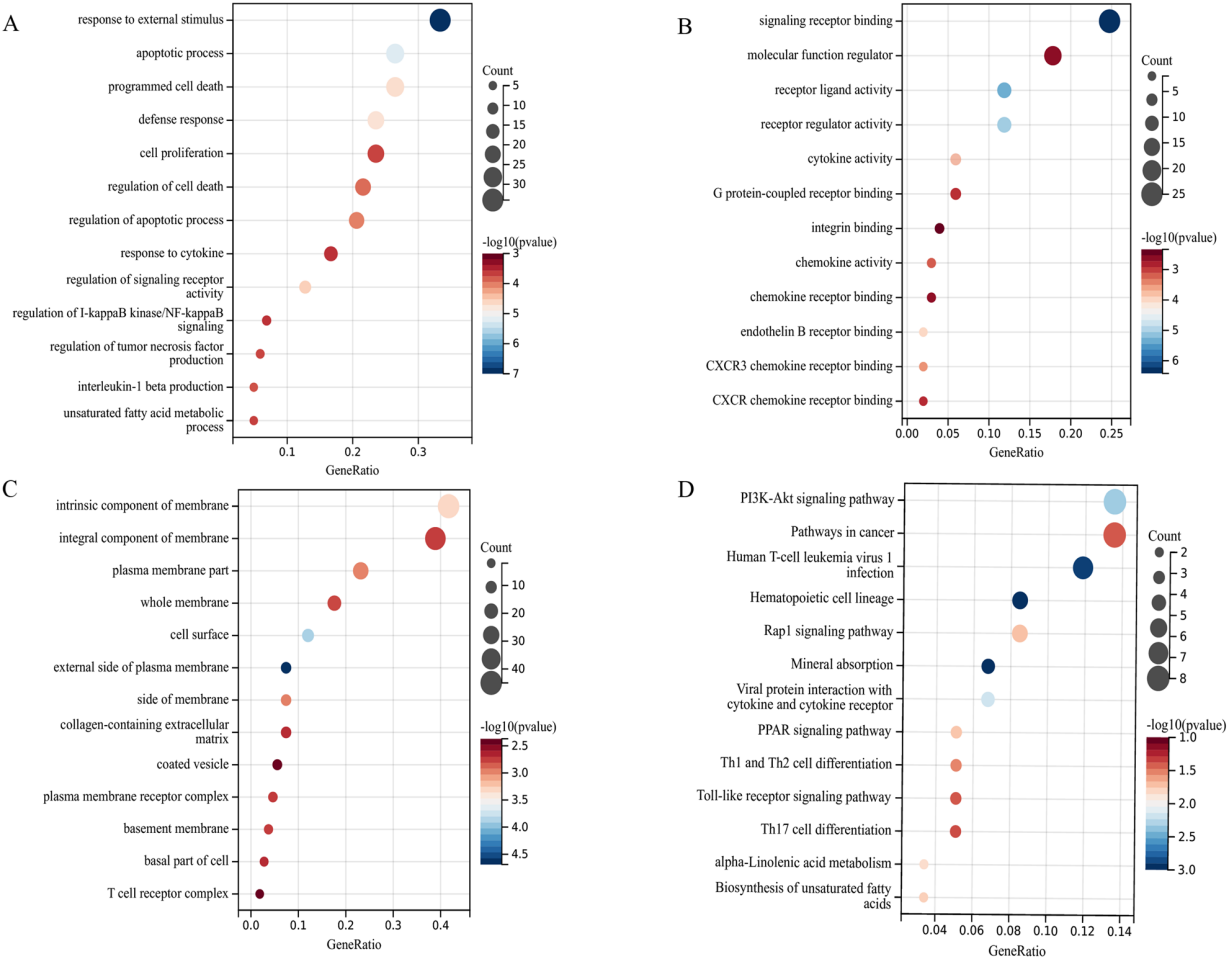
GO function analysis of SDEGs in (**A**) biological progress, (**B**) cellular component, and (**C**) molecular function. (**D**) KEGG pathway analysis of SDEGs. The enrichment significance gradually increases from red to blue, and the dot size represents the number of genes contained in the corresponding pathway.

GO and KEGG pathway enrichment analyses were performed to gain deeper insights into the biological functions of the SDEGs. Following screening using a threshold of *P* value < 0.05, we identified and selected significantly enriched GO terms and KEGG terms (Fig. 3A–D). In the biological process category, SDEGs predominantly participated in crucial processes such as the apoptotic process, cell death, defense response, cytokine response, and regulation of signaling. In the cellular component category, SDEGs displayed notable associations with membrane, plasma, and T-cell receptors. In the molecular function category, SDEGs were mainly involved in receptor-ligand and regulator activity, cytokine and chemokine activity, and chemokine receptor binding. Moreover, the KEGG pathway enrichment analysis revealed that SDEGs were significantly enriched in the pathways of cancer, hematopoietic cell lineage, and multiple signaling pathways, including PI3K-Akt, Rap1, PPAR, and Toll-like receptor signaling pathways.

### PPI network analysis and hub genes selection

**Figure 4:**
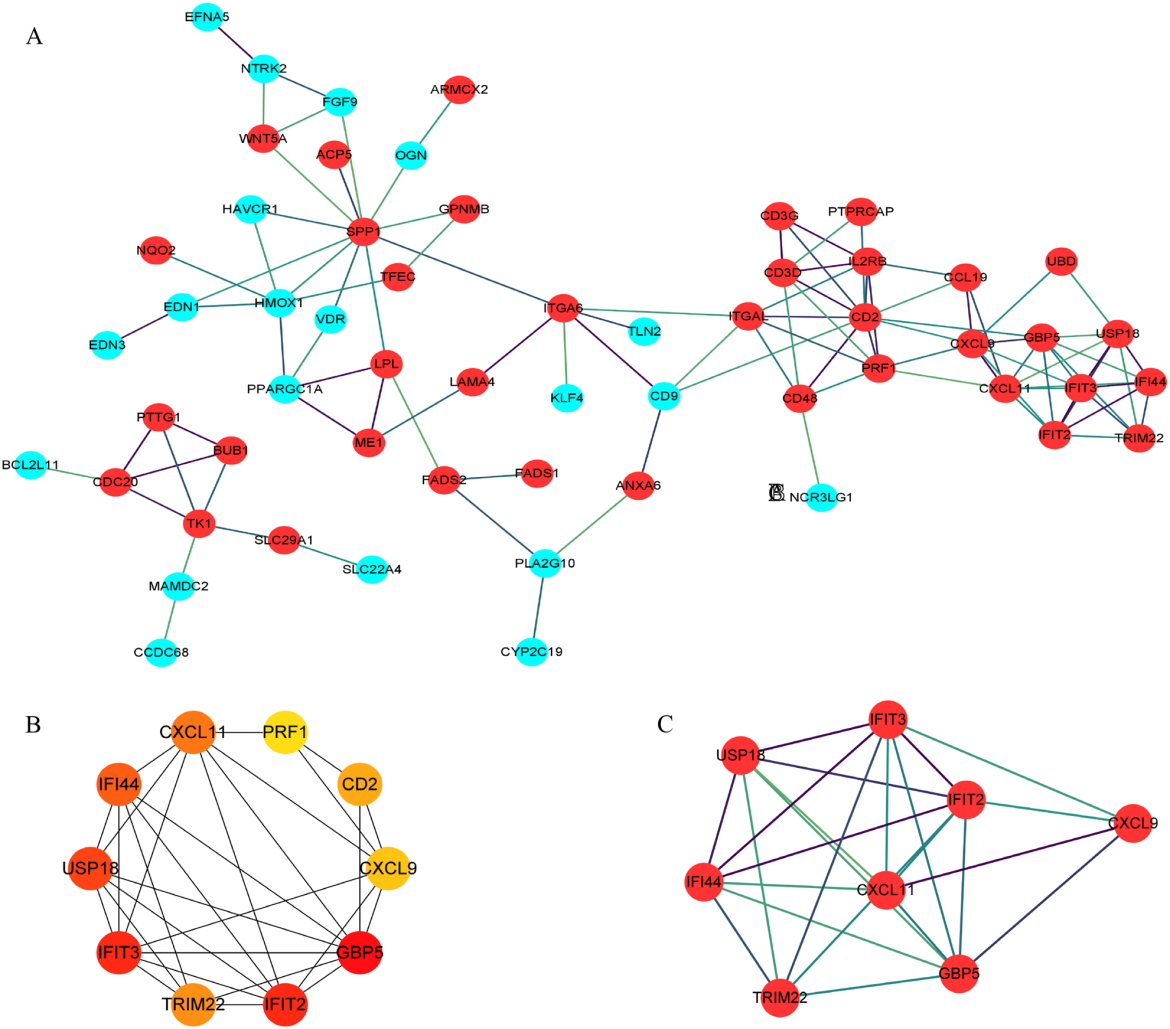
PPI network construction and hub genes identification of SDEGs. (**A**) PPI network of SDEGs. Red and blue circle nodes indicate up- and down-regulated SDEGs, respectively. (**B**) Identification of the top ten hub SDEGs by the MCC algorithm in cytoHubba. The color and size of the circular node depend on the degree of the node. (**C**) The No.1 cluster extracted using the MCODE plug-in.

The 116 SDEGs of NASH and IBD were subjected to analysis within the STRING database. The outcomes of this analysis were then imported into Cytoscape software for visual examination (Fig. 4A). Thereafter, Cytoscape plug-in cytoHubba was utilized to screen out the top 10 of the important genes in the PPI network based on the MCC algorithm, including CD2, PRF1, CXCL11, IFI44, USP18, IFIT3, TRIM22, IFIT2, CXCL9 and GBP5 (Fig. 4B). All 10 hub genes were up-regulated in both NASH and IBD patients. The MCODE plug-in was used to identify significant gene cluster modules from the PPI network. Module 1 network included 8 nodes and 24 edges with a cluster score of 6.857, 8 out of 10 hub genes obtained by cytoHubba were also highlighted in the module 1 network by MCODE (Fig. 4C), which could be key drug targets and biomarkers in NASH and IBD associated with various biological mechanisms.

### Identification of SWDEGs in NASH and IBD via WGCNA

**Figure 5:**
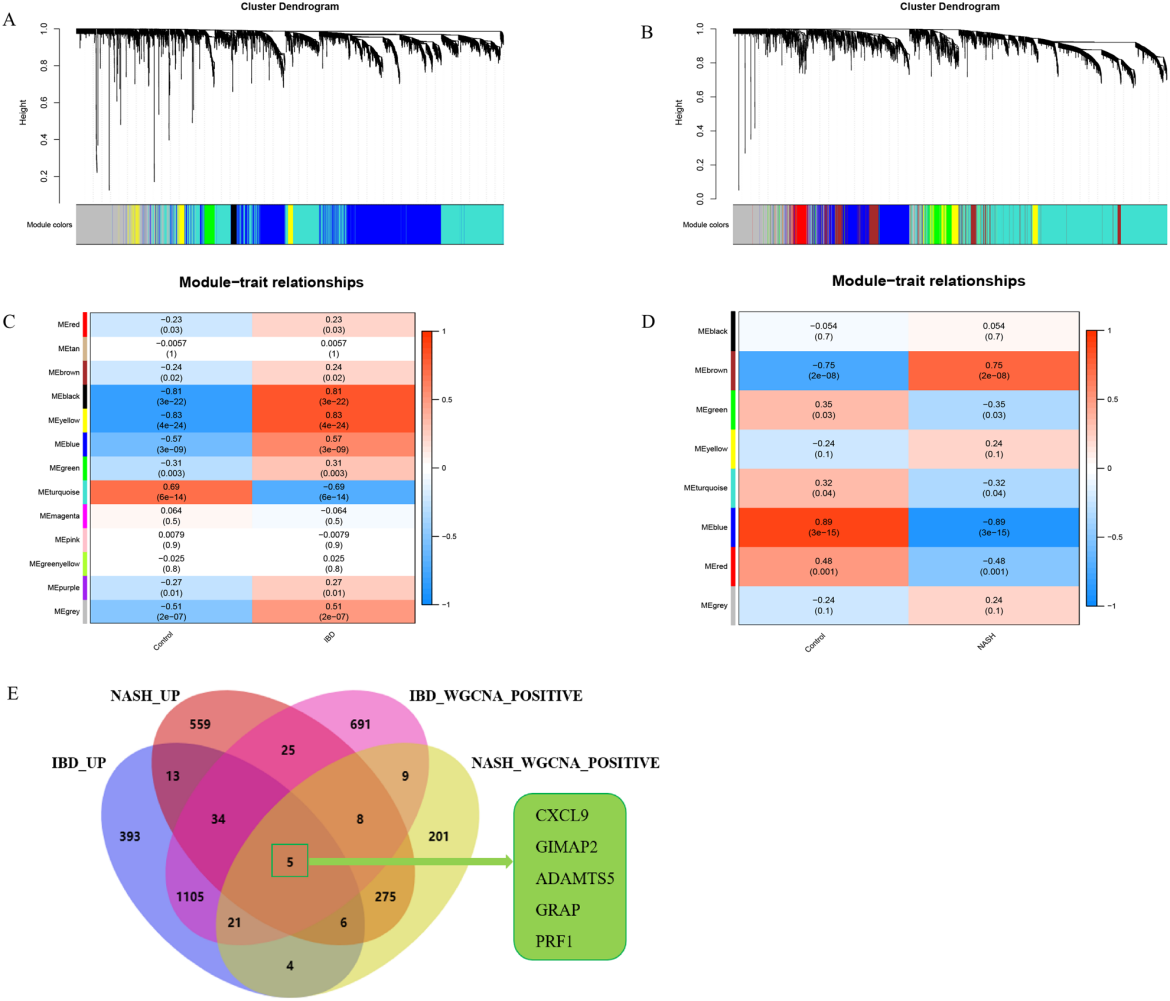
Identification of SWDEGs in NASH and IBD via WGCNA. (**A**) The dendrogram of the co-expressed gene cluster in IBD dataset GSE59071. (**B**) The dendrogram of the co-expressed gene cluster in NASH dataset GSE89632. (**C**) Correlation analysis between expression of module genes and disease phenotypes in IBD dataset GSE59071. (**D**) Correlation analysis between expression of module genes and disease phenotypes in NASH dataset GSE89632. (**E**) Venn diagram of five SWDEGs screened from the intersection of up-regulated SDEGs and gene modules positively related NASH and IBD.

In GSE59071, thirteen distinct modules were identified via WGCNA, each module denoted by a unique color scheme (Fig. 5 A, C). Five modules labeled as ‘MEblack’ (r = 0.81, *P* = 3e−22), ‘MEyellow’ (r = 0.83, *P* = 4e−24), ‘MEblue’ (r = 0.57, *P* = 3e−09), ‘MEgrey’(r = 0.51, *P* = 2e−07) and ‘MEturquoise’ (r = 0.68, *P* = 6e−14) exhibited a high correlation with IBD. Notably, the 'MEblack’, ‘MEyellow’, ‘MEblue’, and ‘MEgrey’ modules demonstrated a positive correlation with IBD, while the ‘MEturquoise’ module displayed a negative correlation. The top three positively correlated modules ‘MEblack’, ‘MEyellow’, and ‘MEblue’ were identified as IBD-related modules, and a total of 1898 genes from the three modules were subsequently subjected to further analyses. Similarly, eight modules from the GSE89632 dataset were identified via WGCNA (Fig. 5B, D), two modules ‘MEbrown’ (r = 0.75, *P* = 2e−08) and ‘MEblue’ (r = 0.89, *P* = 3e−15) exhibited a high correlation with NASH. The ‘MEbrown’ module showed a positive correlation with NASH, while the 'MEblue’ module displayed a negative correlation. The positively correlated module ‘MEbrown’, containing 529 genes, was identified as a NASH-related module.

**Figure 6:**
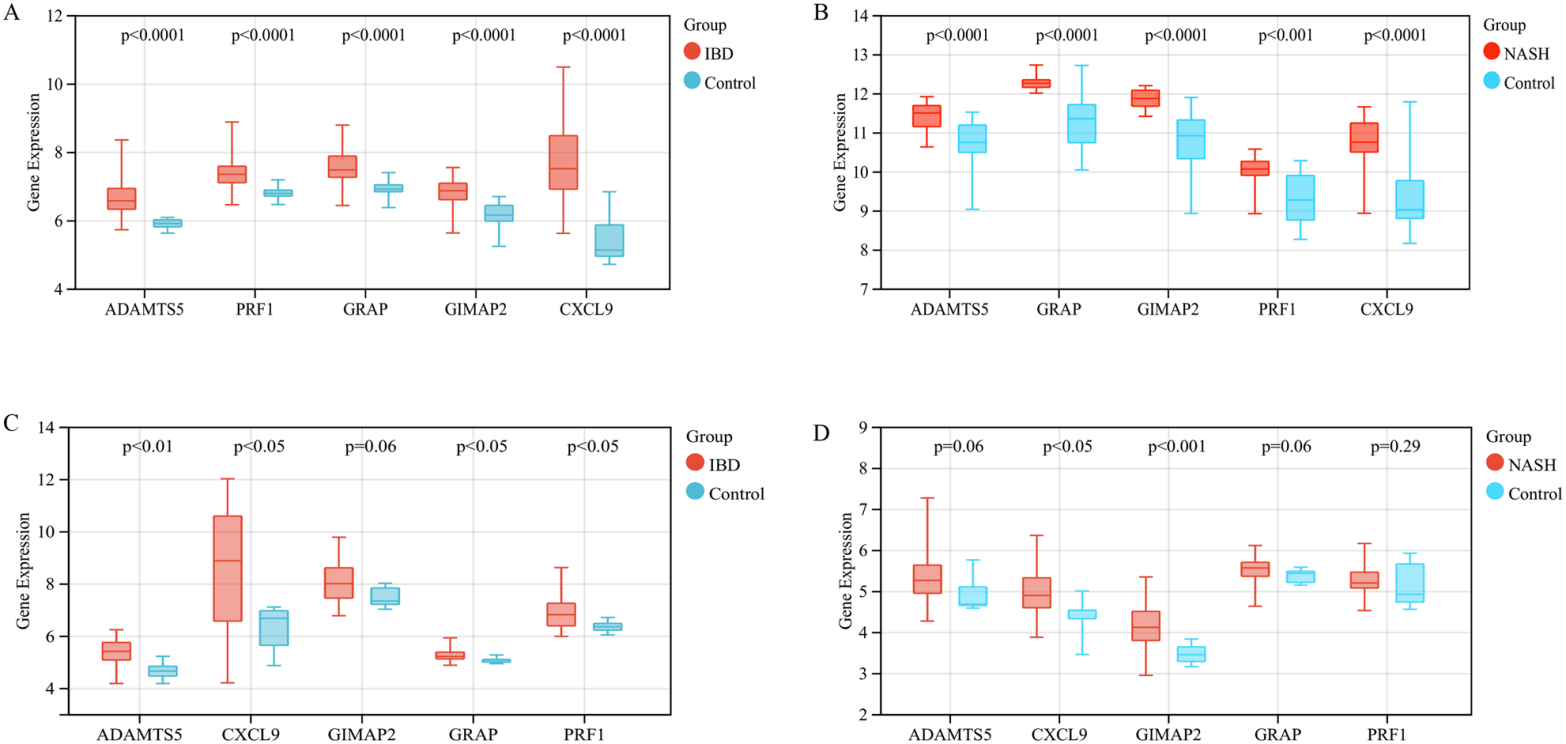
Gene expression level of five SWDEGs in (**A**) GSE59071, (**B**) GSE89632, (**C**) GSE36807, and (**D**) GSE164760.

A total of five SWDEGs (CXCL9, GIMAP2, ADAMTS5, GRAP, and PRF1) were screened from the intersection of SDEGs, IBD positively correlated gene modules (‘MEblack’, ‘MEyellow’, and ‘MEblue’) and NASH positively related gene module (‘MEbrown’) (Fig. 5E). To validate the significance of the five SWDEGs beyond the GSE59071 and GSE89632 datasets, we extended our analyses to additional two datasets—GSE36807 for IBD and GSE164760 for NASH (Fig. 6A–D). The gene expressions of the five SWDEGs within the NASH and IBD groups were consistently elevated in comparison to the control group. This collective exploration underscored the potential involvement of these genes in the pathogenesis of both NASH and IBD.

### Assessment of the diagnostic validity of SWDEGs for NASH and IBD

**Figure 7:**
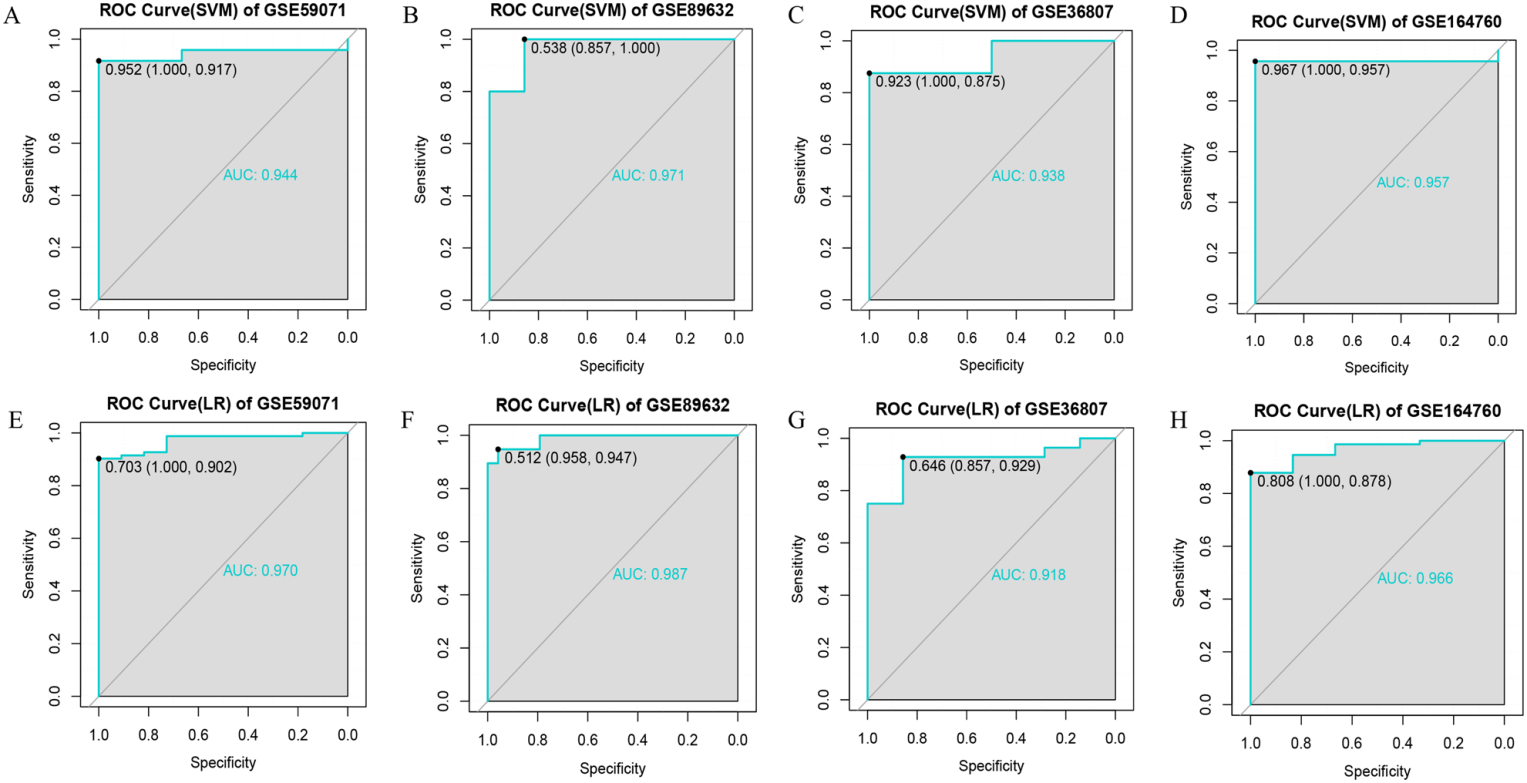
The ROC curves estimating the diagnostic performance of the five-SWDEGs prediction model. (**A**) IBD dataset GSE59071 by SVM algorithm, (**B**) NASH dataset GSE89632 by SVM algorithm, (**C**) IBD validation dataset GSE36807 by SVM algorithm, (**D**) NASH validation dataset GSE164760 by SVM algorithm, (**E**) IBD dataset GSE59071 by LR algorithm, (**F**) NASH dataset GSE89632 by LR algorithm, (**G**) IBD validation dataset GSE36807 by LR algorithm, (**H**) NASH validation dataset GSE164760 by LR algorithm.

Furthermore, the diagnostic efficacy of five SWDEGs was assessed across four datasets (GSE59071, GSE89632, GSE36807, and GSE164760) by constructing different five-SWDEG prediction models based on SVM and LR algorithms. As is shown in Fig. 6, all datasets exhibited high AUC values on the ROC curves using two classifier algorithms. The AUC values of the SVM model in GSE59071, GSE89632, GSE36807, and GSE164760 were 0.944, 0.971, 0.938, and 0.957 respectively (Fig. 7A–D). The AUC values of LR model in GSE59071, GSE89632, GSE36807, and GSE164760 were 0.970, 0.987, 0.918, and 0.966 respectively (Fig. 7E–H), which suggested that high diagnostic potential of five SWDEGs to discriminate between IBD and non-IBD patients, or NASH and non-NASH patients. Besides, each gene of five SWDEGs also showed high AUC values in four datasets of NASH and IBD (Supplementary Fig. 3A–D).

### Prediction of TFs and miRNAs associated with SWDEGs

**Figure 8:**
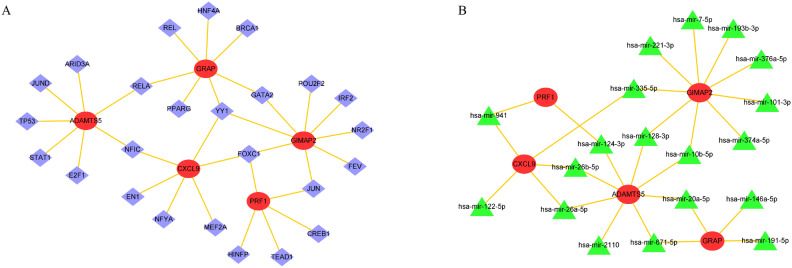
Interaction networks of (**A**) TFs-SWDEGs and (**B**) miRNAs-SWDEGs. The highlighted red ellipse nodes indicate SWDEGs, purple rhombus nodes indicate TFs and blue triangle nodes indicate miRNAs.

To comprehensively elucidate the intricacies of gene expression alterations at the transcriptional level and gain a profound understanding of the regulatory influence exerted by miRNAs on the SWDEGs in NASH and IBD, NetworkAnalyst was utilized to predict the involvement of TFs and miRNAs in interaction with the five SWDEGs and Cytoscape to visualize the TFs-SWDEGs and miRNAs-SWDEGs regulatory network (Fig. 8A,B). In the TF-SWDEGs network, two pivotal TFs (YY1 and FOXC1) exhibited interactions with four SWDEGs (CXCL9, GIMAP2, GRAP, and PRF1). In the miRNA-SWDEGs network, seven miRNAs (mir-26b-5p, mir-26a-5p, mir-124-3p, mir-128-3p, mir-10b-5p, mir-20a-5p, and mir-671-5p) associated with ADMTS5 also exhibited interactions with other four SWDEGs (CXCL9, GIMAP2, GRAP, and PRF1). These seven miRNAs might be crucial for regulating the expression of SWDEGs.

### Correlation between SWDEGs and genes related to key regulatory mechanism

**Figure 9:**
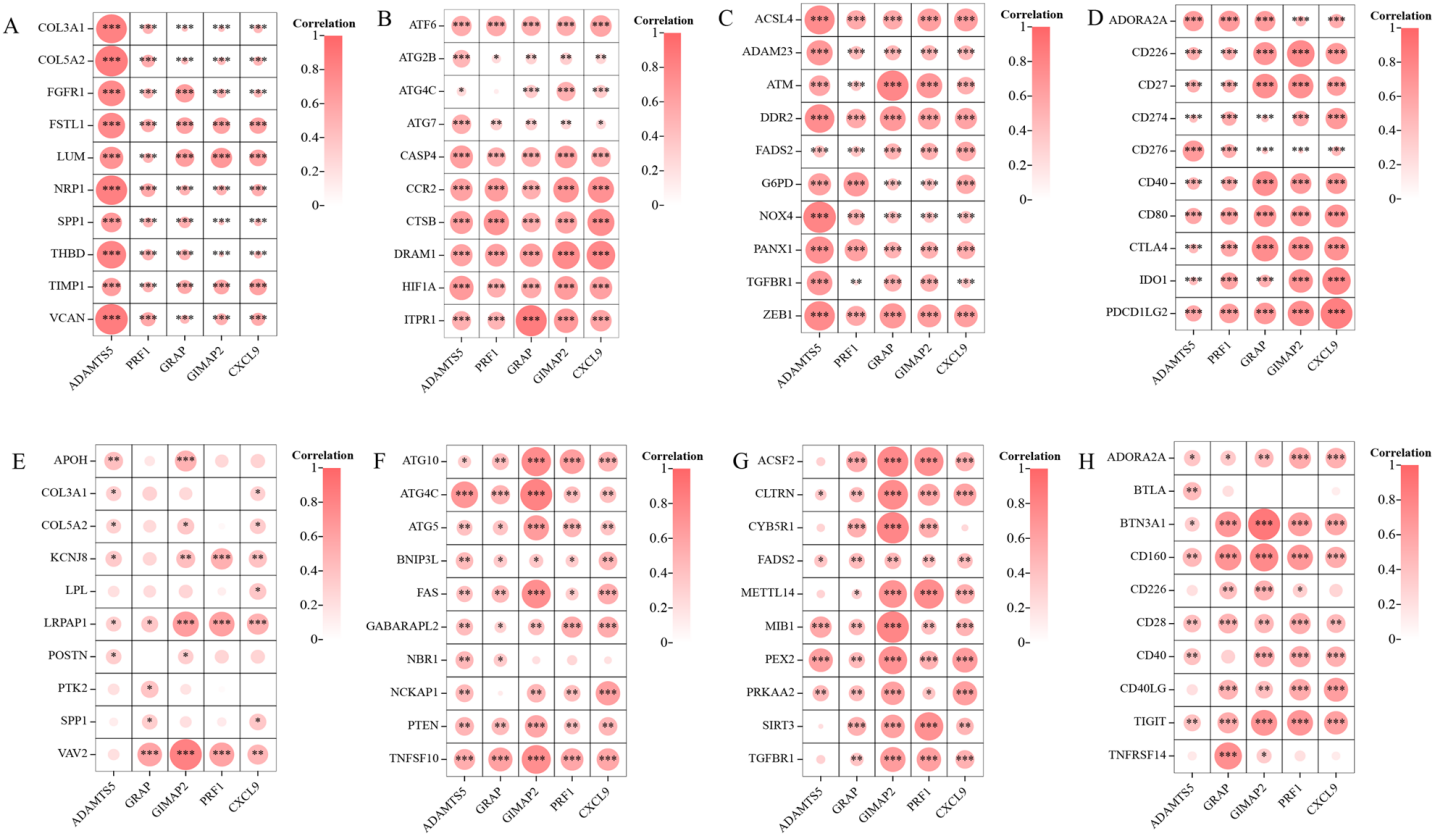
Relationship of SWDEGs and key regulatory genes. (**A**–**D**) Correlation of SWDEGs with angiogenesis-, autophagy-, ferroptosis-, and immune checkpoint-related genes in IBD dataset GSE59071. (**E**–**H**) Correlation of hub genes with angiogenesis-, autophagy-, ferroptosis-, and immune checkpoint-related genes in NASH dataset GSE89632The color and size of the circular node depend on the correlation value. *P < 0.05; **P < 0.01; ***P < 0.001; ns, non-significant.

We performed correlation analysis of four gene sets (FRGs, AURGs, ANRGs, and ICRGs) with five SWDEGs in NASH and IBD respectively, and extracted the top 10 genes with high positive relevance scores in each gene set (Fig. 9A–H). We noticed a stronger correlation between SWDEGs (CXCL9, GIMAP2, ADAMTS5, GRAP, and PRF1) and four gene sets in GSE59071 than that in GSE89632. Moreover, GIMAP2 had a significant correlation with the autophagy-related gene ATG4C in both diseases. For ICRGs, ADORA2A, CD226, and CD40 were correlated with five SWDEGs with different significance in both diseases.

### Analysis of regulatory gene expression in single cells

**Figure 10:**
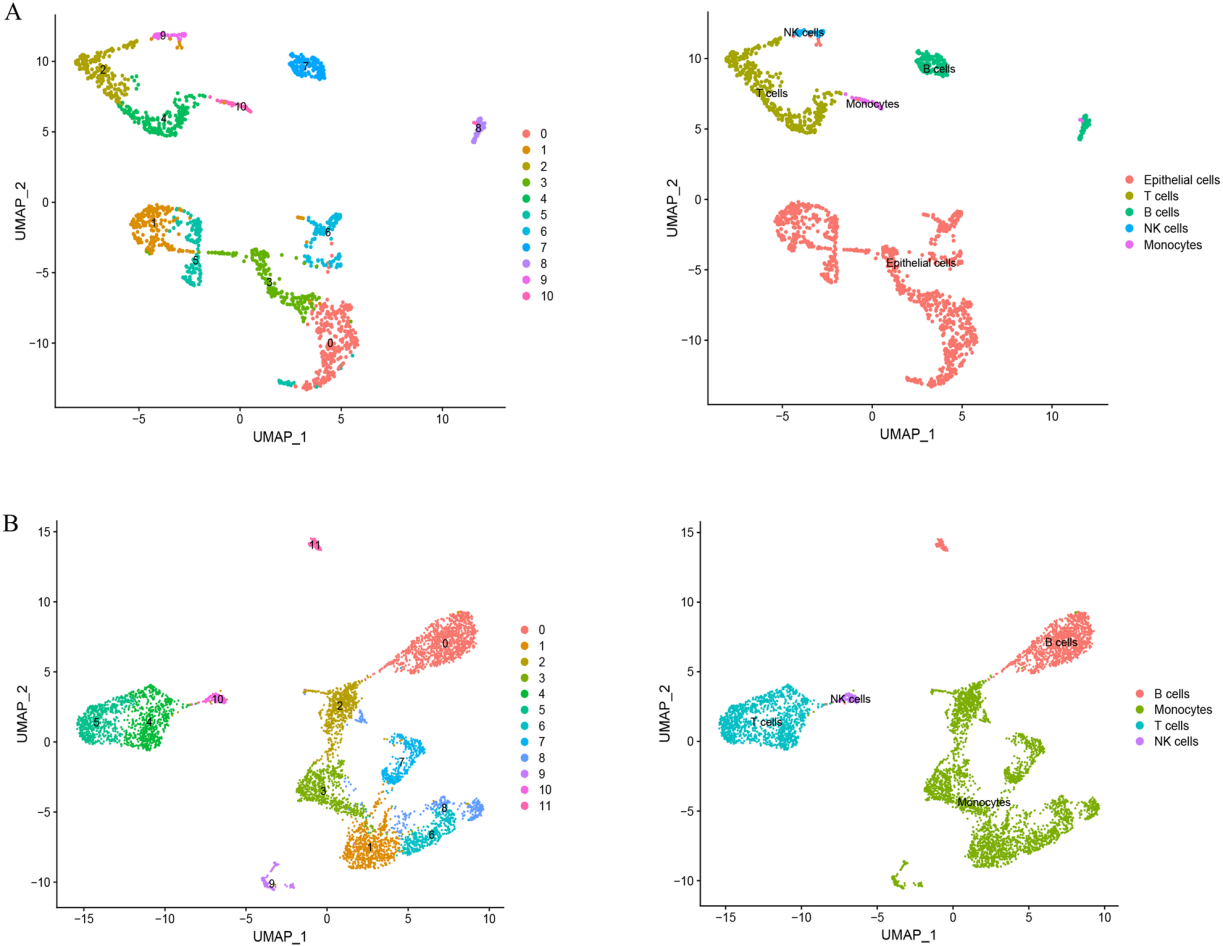
Single-cell analysis of NASH and IBD. (**A**) Cellular subtypes of IBD single-cell sequencing dataset GSE164985 and (**B**) NASH single-cell sequencing dataset GSE19048.

We obtained the two distinct single-cell sequencing datasets, GSE164985 for IBD and GSE190487 for NASH, and subjected them to single-cell analysis utilizing the ‘Seurat’ package. Employing the UMAP algorithm, we performed cellular clustering, and subsequently capitalized on the HumanPrimaryCellAtlasData and BlueprintEncodeData as our primary reference for cellular annotation, each cluster was annotated via the ‘SingleR’ package (Supplementary Table S2). Within the GSE164985 dataset, all cells were classified into five categories: B cells, T cells, epithelial cells, NK cells, and monocytes (Fig. 10A). Similarly, the cells within the GSE190487 dataset were classified into four primary categories: B cells, T cells, NK cells, and monocytes (Fig. 10B).

**Figure 11:**
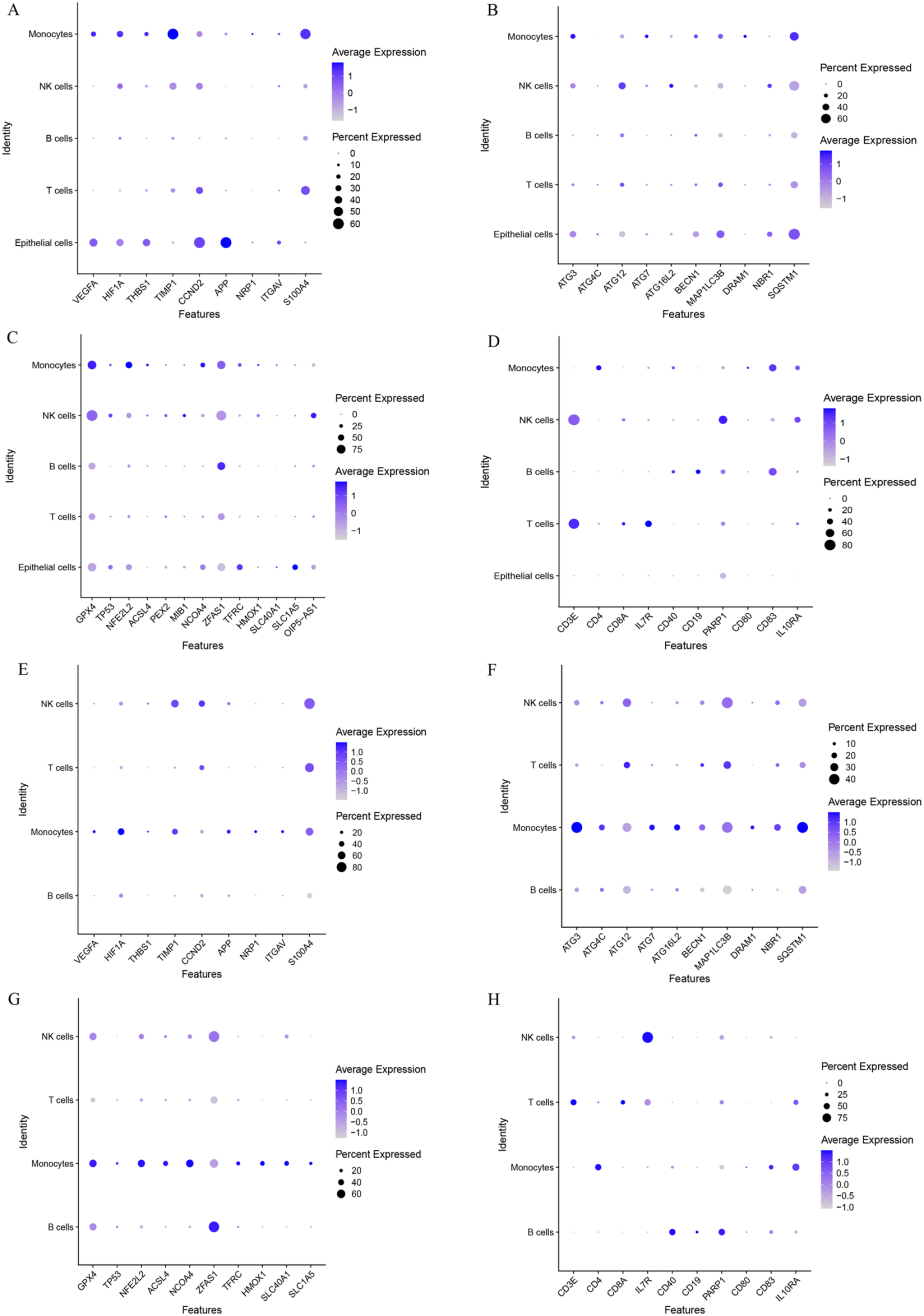
Expression profiles of regulatory genes in single cells of NASH and IBD. (**A**–**D**) Bubble plot of the expression of angiogenesis-, autophagy-, ferroptosis-, and immune checkpoint-related genes in IBD single-cell sequencing dataset GSE164985. (**E**–**H**) Bubble plot of the expression of angiogenesis-, autophagy-, ferroptosis-, and immune checkpoint-related genes in NASH single-cell sequencing dataset GSE19048.

In pursuit of a comprehensive understanding of the cellular landscape within NASH and IBD, we explored the expression levels of genes related to immune responses, ferroptosis, autophagy, and angiogenesis within various cell categories. The results indicated a consistent expression pattern of these regulatory genes across various immune cell subtypes in NASH and IBD (Fig. 11A–H).

### Immunofluorescence analysis of SWDEGs in human liver and colon samples

**Figure 12:**
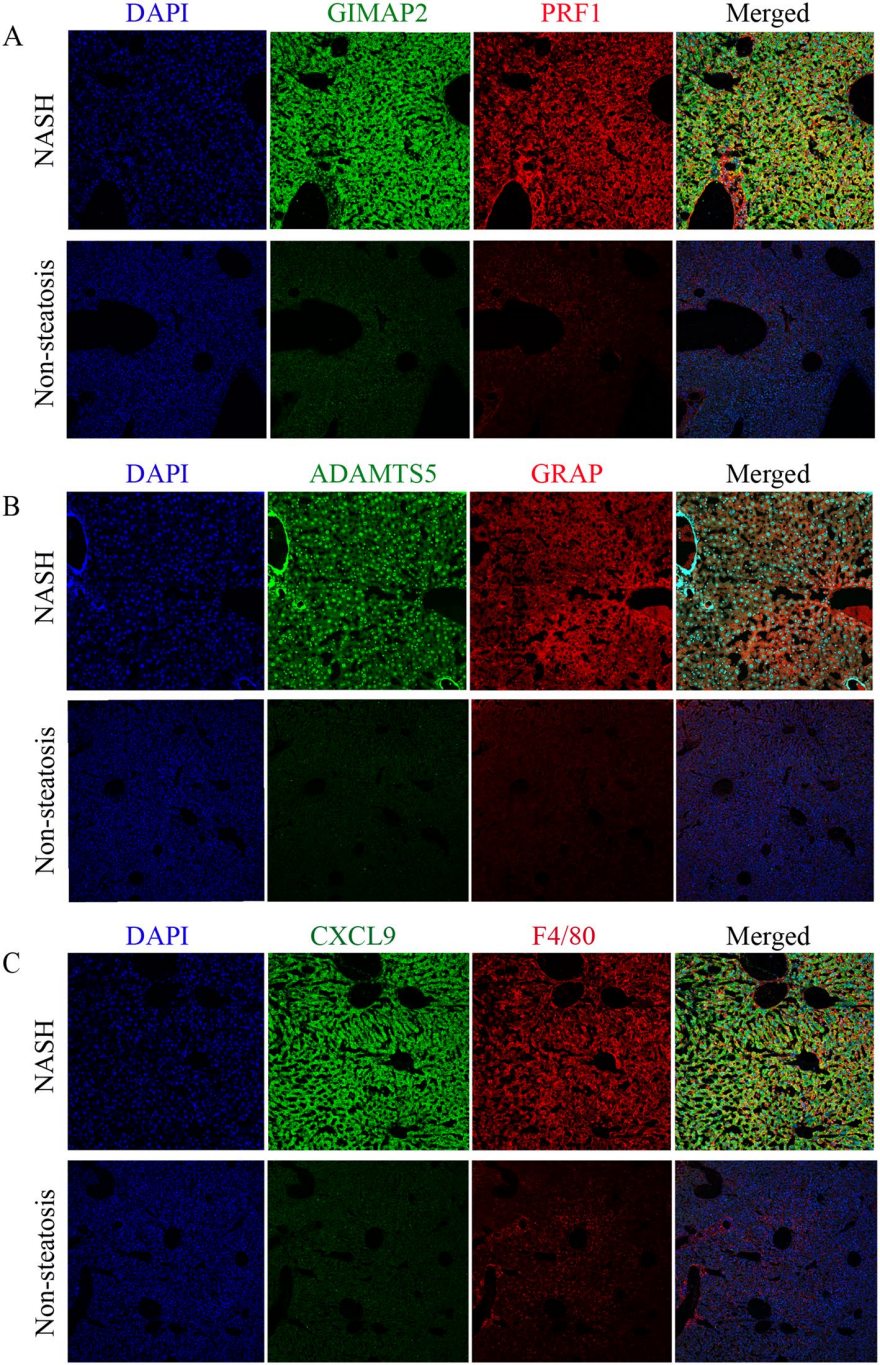
Representative immunofluorescence staining images of five SWDEGs and F4/80 expression in the liver samples of human donors with non-steatosis phenotype and NASH phenotype. (**A**) The co-expression of GIMAP2 and PRF1. (**B**) The co-expression of ADAMTS5 and GRAP. (**C**) The co-expression of CXCL9 and F4/80.

**Figure 13:**
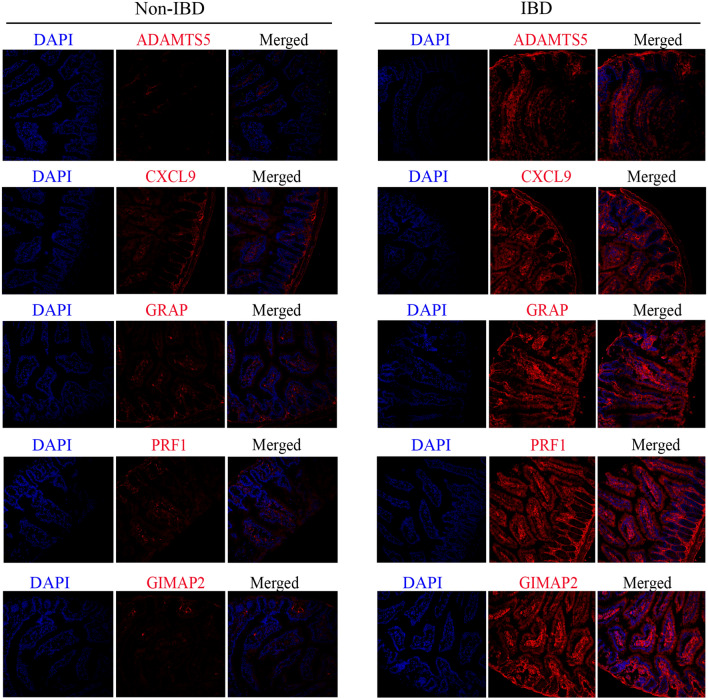
Representative immunofluorescence staining images of five SWDEGs expression in the colon samples of human donors with non-IBD phenotype and IBD phenotype.

To verify the enhanced expression patterns of five SWDEGs in NASH and IBD patients, immunofluorescence analysis was conducted to examine the expression of CXCL9, ADAMTS5, GIMAP2, GRA, PRF1, and F4/80 (a marker for macrophages) in our collected human samples. Notably, the similar results we obtained from bioinformatic analysis were further observed and confirmed in the liver tissues with NASH and non-steatosis phenotype (Fig. 12A–C) and colon tissues with IBD and non-IBD phenotype (Fig. 13).

## Discussion

NASH and IBD are states of chronic inflammation, and the co-existence of the two diseases is becoming increasingly recognized, suggesting the potential presence of shared underlying pathogenic mechanisms and therapeutic targets between them. However, until now, the relationship between NASH and IBD remains unclear, and there is still a lack of effective medical treatments for the two diseases, underscoring the urgent need for the identification and validation of novel biomarkers capable of tracking NASH and IBD progression^40–42^. In this study, we aimed to identify shared signature genes between NASH and IBD for potential biomarker discovery and drug target identification.

Firstly, 116 shared differentially expressed genes (SDEGs) between NASH and IBD datasets were identified through bioinformatics analysis. Then, GO and KEGG pathway enrichment analyses of the SDEGs provided insight into the biological functions and signaling pathways shared by NASH and IBD. Notably, these SDEGs were significantly enriched in biological processes of the apoptotic process, cell death, defense response, cytokine response, and regulation of signaling pathways. Importantly, the enriched signaling pathways, including PI3K-Akt, Rap-1, PPAR, and Toll-like receptor (TLR) signaling, have been closely linked to the development and pathogenesis of NAFLD/NASH and IBD^43–48^. Particularly, the TLR signaling pathway emerged as a prominent signal, leading to the activation of the innate immune system, upregulation of inflammatory cytokines, and activation of downstream inflammatory pathways^49–51^. Similarly, PPAR signaling pathway plays an essential role in regulating gene expression involved in various cellular processes, including lipid metabolism, inflammatory and immune response, cell proliferation, and fibrosis, which has significant effects on the progression of both NASH and IBD^52,53^. Additionally, PPI network analysis identified 10 hub genes within the SDEGs, such as CD2, PRF1, CXCL11, IFI44, USP18, IFIT3, TRIM22, IFIT2, CXCL9, and GBP5, all up-regulated in NASH and IBD patients, underlining their importance in the pathogenesis of two diseases. These hub genes may serve as key regulators of biological processes and potential biomarkers for disease diagnosis and prognosis.

Furthermore, WGCNA was employed to identify co-expressed modules specifically associated with NASH and IBD. The categorization of these modules as IBD-related and NASH-related modules provides insights into the genes specifically linked to each disease. Notably, five co-expressed SWDEGs (CXCL9, GIMAP2, ADAMTS5, GRAP, and PRF1) derived from the intersection of SDEGs and gene modules with positive correlations hold potential as diagnostic biomarkers, with the capability to discriminate patients with NASH and IBD from healthy individuals. Among these, CXCL9 is a chemokine that plays a crucial role in recruiting specific immune cell populations (T cells and natural killer cells) to sites of inflammation, promoting chronic inflammation and immune-mediated tissue damage, which could be a biomarker for NASH and IBD^54,55^. GIMAP2 is a member of the GTPase family and involved in the regulation of apoptotic pathways, immune cell survival, and homeostasis^56^, however, the exact function of GIMAP2 in NASH and IBD is not fully understood. ADAMTS5 has been linked to inflammatory processes in various conditions, its activity could contribute to the release of pro-inflammatory mediators and cytokines, and exacerbate tissue inflammation^57^. Few studies have elucidated that the absence of ADAMTS5 could preserve liver integrity in diet-induced NASH models^58,59^. GRAP is an adaptor protein involved in Ras signaling^60^, it may participate in various signaling cascades triggered by cell surface receptors^61^, potentially influencing immune responses, inflammation, and cellular processes in the two diseases. PRF1 is primarily known for its role in the immune system, specifically in the cytotoxic function of cytotoxic T lymphocytes (CTLs) and natural killer (NK) cells^62,63^. Thus, PRF1-expressing CTLs and NK cells may be involved in immune-mediated cytotoxicity, targeting and killing infected or damaged cells in the condition of two diseases. Notably, CXCL9 and PRF1 were also hub SDEGs in the PPI network and interacted with each other by connecting with the same cytokines and chemokines, such as CXCL11, CXCL12, XCL1 and CCL5 (Supplementary Fig. 4). In addition, the high diagnostic potential of five SWDEGs for NASH and IBD has been validated across four datasets (GSE59071, GSE89632, GSE36807, and GSE164760) by using SVM and LR algorithms, indicating that these SWDEGs could serve as promising therapeutic targets for both diseases.

Moreover, the prediction of transcript factors (TFs) and miRNAs associated with the five SWDEGs through network analysis offered valuable insights into gene expression changes at the transcriptional level and potential regulatory networks that may modulate the expression of SWDEGs in NASH and IBD. From the TFs-SWDEGs network, two TFs (YY1 and FOXC1) showed a high interaction with four SWDEGs (CXCL9, GIMAP2, GRAP, and PRF1). YY1 is associated with inflammation and immune responses^64^, suggesting a potential role in modulating immune-related gene expression during IBD, and it also has been associated with the progression of NAFLD and NASH^65^. FOXC1 has been identified for its role in promoting the invasion and metastasis of HCC through the PI3K/Akt/HIF-1α signaling pathway^66^, and it emerges as a pivotal TF implicated in the pathogenesis of colitis-associated colon cancer (CAC)^67^. In the miRNAs-SWDEGs network, both mir-26a-5p and mir-26b-5p are involved in the suppression of colorectal cancer^68^. Particularly, mir-26a-5p plays a crucial role in regulating fatty acid and cholesterol homeostasis, protecting against the progression of NAFLD^69^. The mir-20a-5 serves as a key regulator in inflammation-driven liver fibrosis^70^, and it is also involved in the prevention of CD development by improving the intestinal epithelial barrier function^71^. Additionally, the other four key miRNAs in this network are mir-124-3p, mir-128-3p, mir-10b-5p, and mir-671-5p, they all play a potential role in immune regulation or affecting the infiltration of immune cells, as reported in previous studies^72–75^. The identified TFs and miRNAs may serve as key regulators of SWDEGs expression in NASH and IBD, presenting opportunities for targeted therapeutic interventions.

According to previous studies, the pathogenesis of both NASH and IBD was associated with autophagy, ferroptosis, angiogenesis, and immune response^76–81^, which are all key regulatory mechanisms in inflammation and immune-related diseases. A correlation analysis of five SWDEGs with FRGs, AURGs, ANRGs, and ICRGs was performed to unravel the underlying mechanisms of autophagy, ferroptosis, angiogenesis, and immune response in the two diseases. The results showed that the expression patterns of SWDEGs in NASH and IBD had distinct degrees of correlation with genes related to autophagy, ferroptosis, angiogenesis, and immune checkpoint, with stronger correlations observed in IBD datasets, suggesting the importance of these regulatory mechanisms in the pathogenesis of IBD. Additionally, three ICRGs (ADORA2A, CD226, and CD40) showed differently significant correlation with five SWDEGs in both diseases, which provided further insights into potential biological interactions and immune regulatory mechanisms underlying NASH and IBD.

Finally, two single-cell sequencing datasets from NASH and IBD samples were downloaded for single-cell annotation analysis. The annotated cell types were mainly B cells, T cells, NK cells, and monocytes in both diseases. These four cell types are all important components of the immune system and play distinct roles in the pathogenesis of NASH and IBD^82–89^, in which FRGs, AURGs, ANRGs, and ICRGs were expressed to varying degrees. Notably, ICRGs were expressed in four cell types, whereas FRGs, AURGs, and ANRGs were mainly expressed in monocytes of two diseases. Monocytes can differentiate into macrophages and play a crucial role in IBD pathogenesis, contributing to tissue damage and inflammation by releasing inflammatory mediators and participating in immune responses^86^. A recent study indicated that monocytes were also involved in fibrogenesis and related to fibrosis progression in NASH^85^. Furthermore, Kotsiliti et al*.* revealed that gastrointestinal B cells contributed to T cell–driven inflammation and aggravated hepatic fibrosis in mice and patients with NASH, providing a potential target in the gut-liver axis for NASH^83^. The annotation of different cell clusters and the expression levels of FRGs, AURGs, ANRGs, and ICRGs provided valuable information about the cellular heterogeneity and potential molecular pathways in the two diseases.

In general, our study provides novel insights into the connection of NASH and IBD through identification of co-expressed gene modules and analysis of regulatory networks involving five SWDEGs with diagnostic potential, and the elevated expression of these five SWDEGs in NASH and IBD patients was also confirmed by our immunofluorescence analysis. Notably, apart from CXCL9, the other four SWDEGs (GIMAP2, ADAMTS5, GRAP, and PRF1) have not been extensively explored their roles in the pathogenesis of NASH and IBD in previous studies, which could be new potential biomarkers and targets for therapeutic interventions of the two diseases. Moreover, the regulatory mechanisms involving autophagy, ferroptosis, angiogenesis, and immune responses were found to play pivotal roles in both diseases, particularly in IBD, suggesting that targeting these mechanisms could offer novel therapeutic strategies for both diseases.

However, some limitations in this study need to be addressed. Firstly, the functional roles of SWDEGs and their regulatory mechanisms need experimental validation. Secondly, although single-cell analysis provides an overview of gene expression in specific cell types, further investigations are needed to elucidate the functional relevance of specific cell subtypes in the pathogenesis of NASH and IBD.

## Conclusion

In conclusion, this study represents the first attempt to examine shared signature genes and potential regulatory mechanisms between NASH and IBD. The findings showed significant implications for understanding the pathogenesis of two diseases and hold promise for the development of novel diagnostic biomarkers and therapeutic targets for NASH and IBD.

### Supplementary Information

Supplementary Figures.

Supplementary Table S1.

Supplementary Table S2.

## Data Availability

The datasets presented in this study can be found in online repositories. The names of the repository/repositories and accession number(s) can be found in the article/Supplementary Material. Further inquiries can be directed to the corresponding authors.
