# Supplementary material for: Exploring shared molecular signatures and regulatory mechanisms in nonalcoholic steatohepatitis and inflammatory bowel disease using integrative bioinformatics analysis
Source: Sci Rep. 2024 May 27;14:12085. doi: 10.1038/s41598-024-62310-w (PMC11130338; doi:10.1038/s41598-024-62310-w)
Supplement: Supplementary file 1 — Supplementary Figures. [file 41598_2024_62310_MOESM1_ESM.docx]

**Supplementary Information**

**Supplementary Figures**


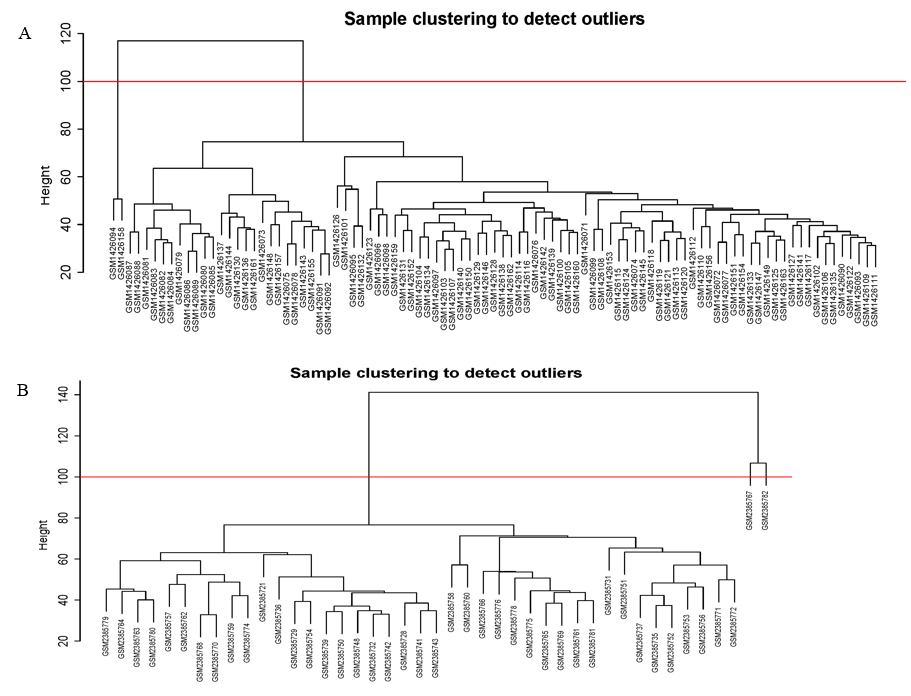


Supplementary Figure 1. Hierarchical clustering analysis of (A) IBD dataset GSE59071 and (B) NASH dataset GSE89632.


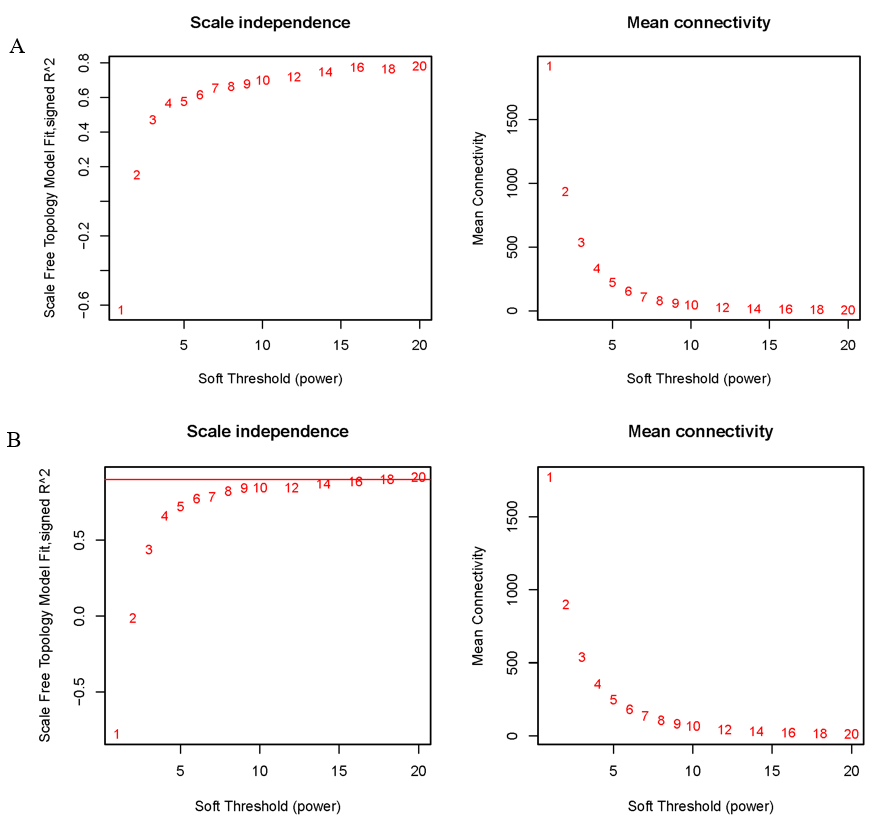


Supplementary Figure 2. The soft thresholds analysis of (A) IBD dataset GSE59071 and (B) NASH dataset GSE89632.


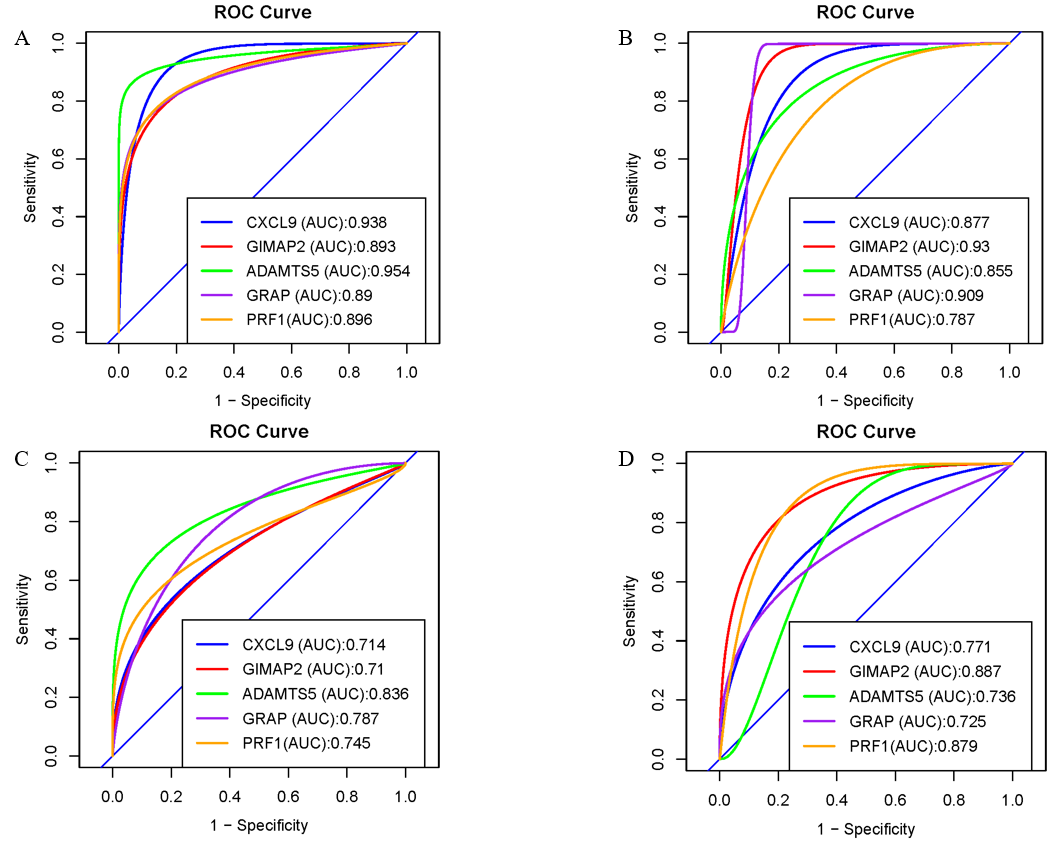


Supplementary Figure 3. The ROC curves of five cWDEGs in (A) IBD dataset GSE59071, (B) NASH dataset GSE89632, (C) IBD validation dataset GSE36807 and (D) NASH validation dataset GSE164760.


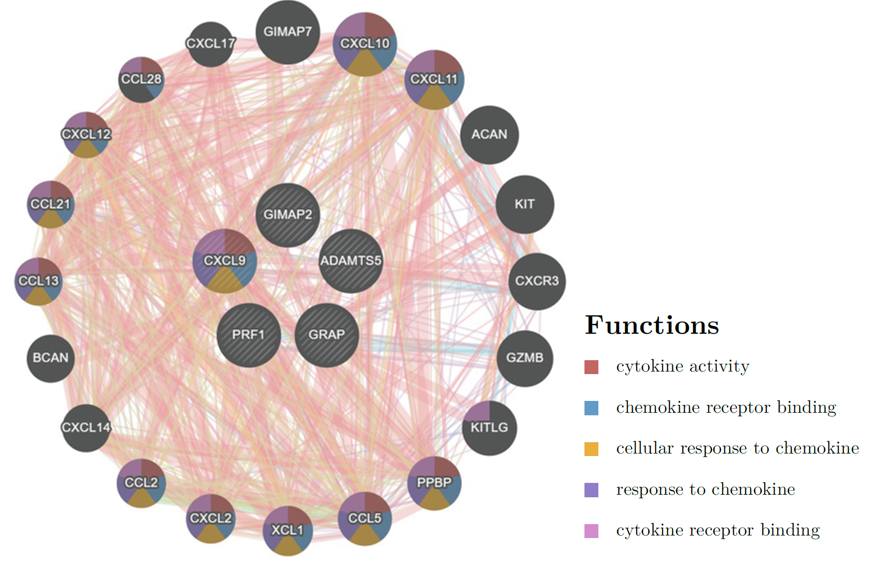


Supplementary Figure 4. PPI network analysis of five cWDEGs by using GeneMania.
